# Influence of implantation of a total hip endoprosthesis on the ipsilateral leg alignment: the effect of sex and dysplasia of the hip

**DOI:** 10.1007/s00402-022-04587-y

**Published:** 2022-08-24

**Authors:** Stefan van Drongelen, Benjamin Fey, Felix Stief, Andrea Meurer

**Affiliations:** 1 Dr. Rolf M. Schwiete Research Unit for Osteoarthritis, Department of Orthopedics (Friedrichsheim), University Hospital Frankfurt, Goethe University Frankfurt, 60528 Frankfurt, Germany; 2 Department of Orthopedics (Friedrichsheim), University Hospital Frankfurt, Goethe University Frankfurt, 60528 Frankfurt, Germany

**Keywords:** Total hip arthroplasty, Developmental dysplasia of the hip, Leg alignment, Sex, Hip osteoarthritis, Femoral offset

## Abstract

**Introduction:**

Differences in leg and hip morphology exist between sexes and developmental hip dysplasia is known to alter proximal femoral morphology. The purpose of this study was to determine whether existing differences in leg alignment due to sex or developmental hip dysplasia have an effect on changes in leg alignment after total hip arthroplasty.

**Materials and methods:**

30 hip osteoarthritis patients underwent biplanar full-length radiography in the standing position preoperatively and 3 months after total hip arthroplasty. Differences in leg alignment between men and women and between patients with primary hip osteoarthritis and patients with developmental dysplasia before and after surgery were tested using a general linear model for repeated measures.

**Results:**

Implantation of a hip prosthesis had no differential effect on ipsilateral leg alignment in patients with hip osteoarthritis due to dysplasia and in patients with primary hip osteoarthritis. However, patients with hip osteoarthritis due to dysplasia had a 2.1° higher valgus both before and after surgery. After total hip arthroplasty, women had a significantly greater increase in varus angle (1.6° vs. 0°) and femoral offset (10.5 vs. 4.6 mm) compared with men. Because the change in acetabular offset was smaller (2.2 vs. 6.2 mm), the global femoral offset was only increased in women. Femoral torsion was constant for men (15.0° and 16.5°), whereas femoral torsion was significantly reduced in women (19.9° and 13.2°).

**Conclusions:**

Hip arthroplasty has a greater effect on leg axis in women than in men. The axial leg alignment of women could change from a natural valgus to a varus alignment. Therefore, surgeons should consider the effects of total hip arthroplasty on leg alignment in patients with hip osteoarthritis. Whether these changes in leg alignment are also clinically relevant and lead to premature medial or lateral knee osteoarthritis should be investigated in future work.

**Trial registration:**

This study was registered with DRKS (German Clinical Trials Register) under the number DRKS00015053. Registered 1st of August 2018.

**Supplementary Information:**

The online version contains supplementary material available at 10.1007/s00402-022-04587-y.

## Background

When total hip arthroplasty (THA) has to be performed because of osteoarthritis (OA), the main goal is to improve the patient's quality of life. In order for the THA to perform its function optimally, the restoration of a normal anatomical leg alignment is a crucial parameter [27]. In addition to the correct implant selection, the correct positioning of the implant also plays an important role [2, 18].

Variations in leg alignment exist in the normal population. It is known that there are differences between the sexes: due to differences in pelvic geometry, women have a more valgus leg, a smaller femoral offset (FO), and a larger neck–shaft angle (NSA) compared with men [3, 12, 33, 34]. It has been suggested that these sex-related differences in lower limb alignment should be considered in the analysis and surgical planning of arthroplasty [11]. Leg alignment may also change due to pathology. Developmental dysplasia of the hip is one of the most common prearthrotic deformities [16]. The dysplasia alters the morphology of the proximal femur [10, 39] and, consequently, the position of the natural hip rotation center, which surgeons try to restore during THA. It is a fact that the distorted anatomy causes major problems in THA [45]. Women are more likely to suffer from dysplasia compared with men [25].

In THA surgery, the FO is increased to reconstruct the global femoral offset (GO). Several studies have shown that a larger FO provides more stability, reduces wear [23, 36], and provides a better functional outcome [14]. A larger FO can enlarge the genu varum [44]. The altered alignment may change the dynamic load distribution on the knee compartments to the extent that knee OA is induced in the medial compartment [21, 22]. However, FO does not take into account the acetabular cup positioning. After reaming; the acetabular offset (AO) is reduced; resulting in a mild medialization. Therefore, the AO, the distance from the center of the femoral head to the perpendicular line passing through the medial edge of the ipsilateral teardrop [8, 26], is added to the FO to obtain the GO [28]. Mahmood et al. [28] showed that a reduction in GO of more than 5 mm was associated with less abductor muscle strength.

The preferred diagnostic tool for verifying the planning and proper fit of prostheses is a conventional 2D pelvic overview radiograph taken with the patient in a supine position. Leg alignment parameters captured in 2D are prone to incorrect positioning of the leg and hip. Small rotational deviations can already lead to significant deviations in alignment [19, 31]. The EOS^®^ system produces biplanar images of the entire lower extremities from the pelvis to the feet with the patient in an upright, standing position [43]. Reconstruction of a 3D image from the EOS images eliminates the problem of erroneous rotations. Radiological 3D parameters of the entire leg can be calculated from these images to verify the fit of the prosthesis and to document changes in radiological leg parameters after THA.

The aim of this study was to determine whether existing differences in leg alignment due to sex or developmental dysplasia of the hip have an influence on changes in leg alignment after implantation of a hip endoprosthesis. Two hypotheses were formulated: (1) women will have a greater increase in femoral offset after THA and thus have a greater increase in varus angle compared to men; (2) patients with hip OA due to dysplasia have a greater increase in femoral offset due to THA, resulting in a greater increase in varus alignment than patients with primary hip OA.

## Methods

### Study design and protocol

In this prospective study, all participating patients with unilateral hip OA underwent biplanar radiography with the EOS^®^ system in the standing position preoperatively and 3 months after THA. This study was carried out from May 2016 till February 2019.

### Patients

Thirty patients (12 men, 18 women) with primary or secondary hip OA who were scheduled for unilateral THA participated in the study. Because this study was part of a large prospective study, the exclusion criteria included inflammatory arthritis, orthopedic surgery within the past 6 months, previous lower extremity joint replacement, and inability to walk without walking aids. Patients were divided into those with secondary hip OA due to dysplasia and primary hip OA. Dysplasia was present if the center–edge angle was less than 35° [42]. All participants gave written informed consent prior to participation. The protocol was approved by the Medical Ethics Committee of the Department of Medicine, Goethe University Frankfurt (reference number 497/15).

### Radiographic measurements

**Fig. 1:**
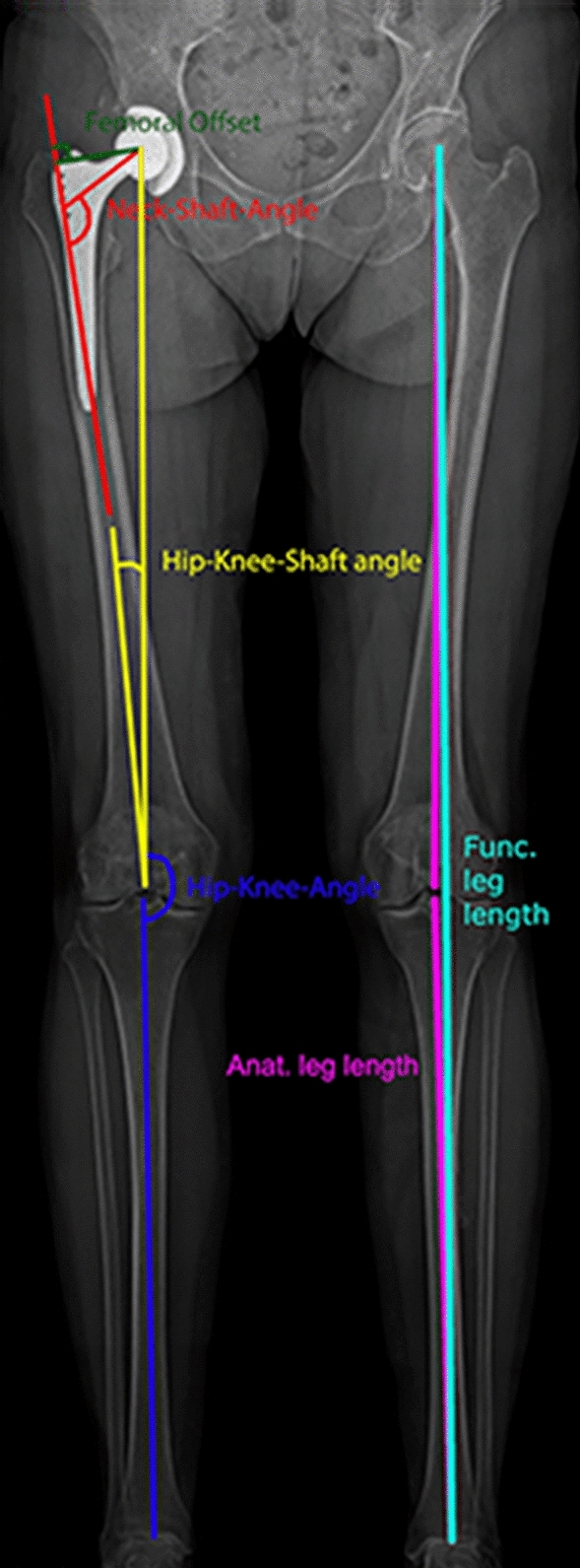
Schematic illustration of the leg alignment parameters projected on a postoperative EOS image. The figure is adapted from [41]

Biplanar radiographs in the standing position were obtained with the EOS^®^ system (EOS imaging, SA, Paris, France) for all patients preoperatively and 3 months postoperatively [7, 13]. A 3D model of the lower extremities was reconstructed from the lateral and anterior images for each patient using sterEOS^®^ (EOS imaging, SA, Paris, France) [15]. Clinical leg parameters were extracted from the 3D reconstruction (Fig. 1).

– Femoral length: distance between the center of the femoral head and the center of the trochlea.
– Functional leg length: distance between the center of the femoral head and the center of the distal articular surface of the tibia.
– Anatomic leg length: sum of femoral length and tibial length (distance between the center of the tibial plateau and the center of the distal articular surface of the tibia).
– Femoral offset (FO): distance between the center of the femoral head and the orthogonal projection of this point on the anatomic axis of the femur.
– Neck–shaft angle (NSA): angle measured between the axis passing from the center of the femoral head through the femoral neck and the line drawn through the center of the femoral diaphysis.
– Hip–knee angle (HKA): angle in the frontal femoral plane between the mechanical axes of the femur and tibia (the line from the center of the tibial plateau to the center of the distal articular surface of the tibia). Valgus > 180°, varus < 180°.
– Hip–knee–shaft angle (HKS): measured in the frontal plane considering the femoral mechanical axis (connecting the centers of the femoral head and trochlea) and the femoral anatomical axis (axis from the center of the trochlea to the center of the distal diaphysis of the femur).
– Femoral torsion/stem torsion: angle defined by the femoral neck axis / femoral stem axis and the posterior condylar axis (axis between the most posterior points of the medial and lateral condyles).

The AO is an important parameter for the description of the natural acetabular position as well as for the postoperative evaluation of the acetabular positioning. The AO is not included in the clinical output of sterEOS. Due to the static pelvic reference point, the AO is less prone to error, so that this parameter was measured in 2D using mediCad (mediCad^®^, mediCAD Hectec GmbH, Landshut, Germany).

**Fig. 2:**
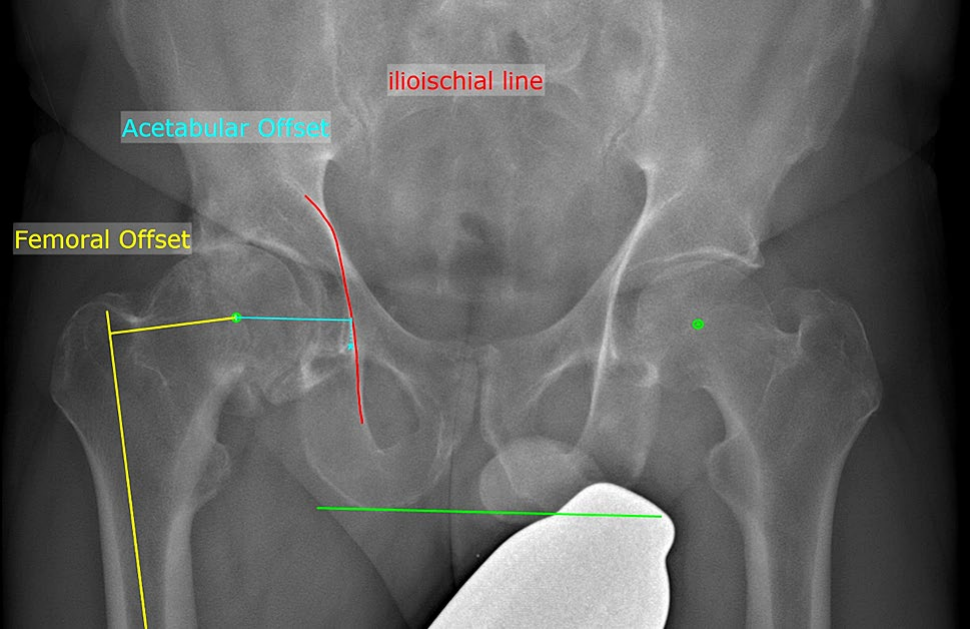
Schematic illustration of the measurement of the femoral and acetabular offset on the frontal EOS image

– Acetabular offset (AO): distance between the center of the femoral head and a perpendicular line passing through the medial edge of the ipsilateral acetabular teardrop. The measurement is made parallel to a line connecting the ipsilateral and contralateral ischium in the frontal plane (Fig. 2).
– Global femoral offset (GO): sum of the femoral offset and the acetabular offset.

### Statistics

Data analysis was performed with SPSS Statistics (IBM SPSS Statistics for Windows, version 26, IBM Corp., Armonk, NY, USA). Shapiro–Wilk tests and visual inspection of Q–Q plots were used to check for normal distribution. Anthropometric data were normally distributed; therefore, differences in anthropometric data between OA types and between sexes were examined with independent sample Students’ *t* tests. A chi-squared test was used to compare the sex distribution between groups (primary and secondary OA). For all analyses, *P* values ≤ 0.05 were considered significant.

Radiographic leg alignment parameters were analyzed for differences between the affected/operated side in the entire patient group using paired-samples Students’ *t* tests or Wilcoxon signed rank test when data were not normally distributed. Differences between groups over time (before and after surgery) were tested using a general linear model (GLM) for repeated measures (within-subject factor, time; between-subject factor, group).

## Results

### Patients

Of the 30 patients, data from three patients could not be included: in two patients, the preoperative EOS image and in one patient, the postoperative EOS image could not be reconstructed because of motion artifacts. The characteristics of the 27 included patients are listed in Table 1. Nine patients were treated for secondary hip OA due to dysplasia, whereas the other 18 patients had primary hip OA. The patients with dysplasia-related hip OA were significantly younger compared with the other patients (Table 1), no differences were observed in height, weight, and body mass index (BMI).

**Table 1:** Anthropometric data of the patients

	Patients (*N* = 27)	Primary OA (*N* = 18)	Dysplasia OA (*N* = 9)	*P* values
Age (years)	62.5 (10.5)	65.8 (8.3)	55.9 (11.6)	**0.017**
Height (m)	1.70 (0.10)	1.69 (0.10)	1.73 (0.10)	0.310
Weight (kg)	82.1 (16.0)	79.6 (17.0)	87.2 (13.2)	0.249
BMI (kgm^−2^)	28.3 (4.5)	27.8 (4.9)	29.1 (3.7)	0.487
Sex (M/F)	12/15	8/10	4/5	1.000

Values are mean values with standard deviation in parenthesis. Comparison between dysplastic hip osteoarthritis patients and primary hip osteoarthritis patients with corresponding *P* values (Independent-sample *t* tests/chi-squared test). *BMI* body mass index; significant differences are bold printed

Because one research question focused on sex differences, characteristics between men and women were also tested: Men were significantly taller and heavier and, accordingly, had a significantly higher BMI.

### Radiological leg alignment parameters

#### General change over time in the whole group of patients

**Fig. 3:**
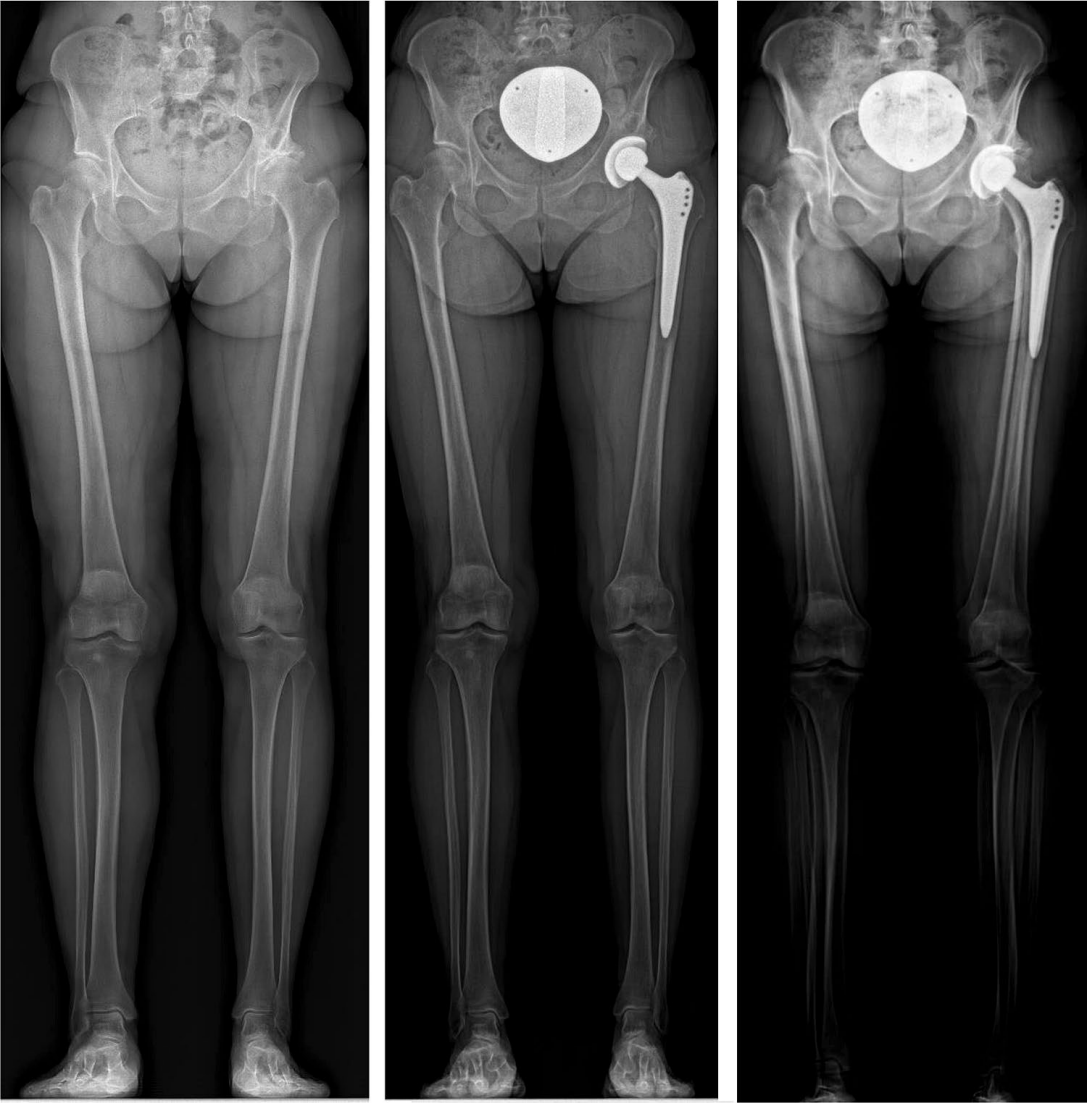
Preoperative, postoperative and overlay of the preoperative and postoperative frontal EOS image showing the decrease in the hip–knee angle, i.e., the increased varus position after surgery, in a 61-year-old woman. The hip–knee angle decreased from 184.3° to 181.8°

After THA, the operated leg showed significant changes in leg alignment. A larger FO (mean increase of 8.0 ± 7.8 mm), a smaller AO (– 4.0 ± 4.3 mm) and as a consequence a larger GO (3.9 ± 8.2). Further, a decreased HKA (– 1.1 ± 1.4°), i.e., a greater varus position of the leg (Fig. 3), and a larger HKS (1.1 ± 0.8°) were calculated. The increase in femoral length resulted in an increase in functional and anatomic leg length of the operated leg after THA.

#### Primary vs. secondary hip OA due to dysplasia

The hip arthroplasty had no differential effect on ipsilateral leg alignment in patients with dysplasia-related hip OA and in patients with primary hip OA (Table 2). A borderline significant difference was found for HKA between OA types (*P* = 0.056). Patients with dysplasia hip OA had greater HKA (2.1°) before and after surgery than the other patients. GLM showed no significant effect for the whole group of patients besides the above-mentioned changes over time. No interaction between time and type of OA was found either.

**Table 2:** Radiological leg alignment parameters for the affected/operated side for patients with primary hip osteoarthritis and patients with hip osteoarthritis due to dysplasia and the results of the general linear model

	Primary OA (*N* = 18)		Dysplasia OA (*N* = 9)		GLM		
	Preoperative	Postoperative	Preoperative	Postoperative	*T*	*O*	*T* × *O*
Femur length (cm)	42.5 (2.9)	43.2 (2.9)	44.4 (2.9)	45.2 (2.9)	**< 0.001**	0.113	0.798
Func. leg length (cm)	79.7 (5.6)	80.6 (5.7)	82.7 (6.0)	83.6 (5.9)	** < 0.001**	0.207	0.983
Anat. leg length (cm)	79.5 (5.7)	80.3 (5.7)	82.3 (5.9)	83.0 (5.9)	**< 0.001**	0.247	0.876
FO (mm)	40.1 (7.7)	46.9 (6.2)	39.8 (8.6)	49.8 (7.5)	**< 0.001**	0.624	0.285
AO (mm)	33.2 (5.1)	29.3 (3.4)	34.3 (5.5)	30.2 (3.0)	**< 0.001**	0.502	0.902
GO (mm)	73.3 (11.3)	76.2 (8.5)	74.1 (13.1)	80.1 (7.7)	**0.014**	0.546	0.366
NSA (°)	126.8 (6.4)	126.9 (7.6)	129.3 (6.3)	127.3 (6.4)	0.498	0.557	0.444
HKA (°)	178.3 (2.7)	177.4 (2.4)	180.4 (3.0)	179.5 (2.5)	**0.002**	0.056	0.847
HKS (°)	5.1 (1.3)	6.1 (1.1)	4.2 (1.3)	5.5 (1.1)	**< 0.001**	0.117	0.364
Torsion (°)	15.7 (10.1)	13.8 (10.9)	21.8 (10.0)	16.5 (14.0)	0.118	0.273	0.459

Values are mean values with standard deviation in parenthesis

*OA* Osteoarthritis, *GLM* general linear model, *FO* femoral offset, *AO* acetabular offset, *GO* global femoral offset, *NSA* neck–shaft angle, *HKA* hip–knee angle, *HKS* hip–knee–shaft angle, *T* time, *O* type of osteoarthritis, *T* × *O* interaction between time and type of osteoarthritis

Significant differences are in bold

#### Men vs. women

Because of the differences in body size (and body composition), all parameters of femoral morphology showed a significant difference between men and women except for HKS and femoral/stem torsion (Table 3). However, the main question was whether the leg axis changed to a similar extent in men and women after surgery. The GLM results showed that this was not the case because of significant interaction effects between sex and time: women had a significantly greater increase in HKA (1.6° vs. 0°), a greater increase in FO (10.5 vs. 4.6 mm), a smaller decrease in AO (– 2.2  vs. 6.2 mm), an increase in GO (8.3 vs. – 1.6 mm) and a greater increase in HKS angle (1.5° vs. 0.7°). In addition, femoral torsion changed differently: while it was constant in men (15.0° and 16.5°), a significant decrease in femoral torsion was observed in women (19.9° and 13.2°).

**Table 3:** Radiological leg alignment parameters for the affected/operated side for men and women and the results of the general linear model

	Men (*N* = 12)		Women (*N* = 15)		GLM		
	Preoperative	Postoperative	Preoperative	Postoperative	*T*	*S*	*T* × *S*
Femur length (cm)	45.3 (2.1)	46.0 (1.8)	41.5 (2.6)	42.2 (2.6)	**< 0.001**	** < 0.001**	0.796
Func. leg length (cm)	84.9 (3.9)	85.9 (3.8)	77.4 (4.8)	78.1 (4.8)	**< 0.001**	**< 0.001**	0.415
Anat. leg length (cm)	84.8 (4.0)	85.5 (3.8)	77.0 (4.7)	77.7 (4.7)	**< 0.001**	**< 0.001**	0.582
FO (mm)	46.6 (5.5)	51.2 (6.1)	34.7 (4.8)	45.3 (6.0)	**< 0.001**	**< 0.001**	**0.037**
AO (mm)	38.0 (2.8)	31.8 (2.7)	30.0 (3.6)	27.8 (2.5)	**< 0.001**	**0.014**	**< 0.001**
GO (mm)	84.6 (5.3)	83.0 (7.2)	64.7 (6.5)	73.1 (6.3)	**0.015**	**< 0.001**	**0.001**
NSA (°)	122.5 (4.5)	122.8 (6.2)	131.7 (4.5)	130.4 (6.0)	0.705	** < 0.001**	0.556
HKA (°)	176.9 (2.0)	176.8 (2.3)	180.7 (2.4)	179.2 (2.4)	** < 0.001**	**0.001**	**< 0.001**
HKS (°)	5.3 (1.2)	6.0 (1.0)	4.3 (1.4)	5.8 (1.2)	**< 0.001**	0.230	**0.005**
Torsion (°)	15.0 (10.4)	16.5 (11.6)	19.9 (10.0)	13.2 (12.1)	0.200	0.844	**0.048**

Values are mean values with standard deviation in parenthesis

*GLM* general linear model, *FO* femoral offset, *AO* acetabular offset, *GO* global femoral offset, *NSA* neck–shaft angle, *HKA* hip–knee angle, *HKS* hip–knee–shaft angle, *T* time, *S* sex, *T* × *S* interaction between time and sex

Significant differences are in bold

## Discussion

This study addressed the question of the effect of THA on the overall leg axis, particularly when preexisting differences in leg alignment due to sex and etiology of hip OA are considered. In the present study, the effect of THA on the leg axis was found to be greater in women than in men, whereas no differences were found between patients with primary hip OA and hip OA due to dysplasia.

This study was performed using the EOS^®^ system, and the leg alignment parameters were calculated in 3D based on a full-length upright standing image, in contrast to the study of Akiyama et al. [1] and Zhao et al. [46], which measured leg alignment in 2D. The upright standing position allows accurate recording of the patient's entire weight-bearing leg. Previous studies have found that measurement errors due to leg positioning are minimized using the EOS^®^ system [40, 43].

Comparison of leg axis before and after THA is not new; van Drongelen et al. [41] found smaller HKA (more varus), larger FO, and larger HKS angle. Similar results, more varus and a larger FO after THA, were also found by Oliver et al. [35]. However, these studies only reported differences in leg alignment after THA and did not consider preoperative differences in leg alignment due to sex or dysplasia.

In the present study, no differences in leg alignment were observed between patients with primary and secondary hip OA. Both groups showed the same changes over times as found for the entire group of patients. The only noticeable abnormality was that the patients with hip OA due to hip dysplasia had a more valgus knee before and after surgery. In contrast to the present study, Sato et al. [37] found a smaller FO in patients with secondary hip OA compared with patients with primary hip OA. Zhao et al. [46] studied only dysplasia patients who received THA and reported that the valgus tendency was significantly reduced after THA, while Cassidy et al. [6] noticed that patients with dysplasia had more increased FO after surgery than patients with other diagnoses. These findings are consistent with the present results, but not only for the dysplasia patients but also for the patients with primary hip OA.

Several studies have been performed on the differences in leg alignment between men and women, independent of hip OA and performance of THA. In the present study, dissimilarities in FO, AO, GO, NSA, and HKA were found in addition to differences in longitudinal measurements, which can be attributed to body size. The preoperative differences between men and women do not explain the variations in FO, AO, GO, HKA and HKS changes between men and women after surgery. This was also noted by Degen et al. [11], who concluded that sex-related differences should be considered in analysis and surgical planning. However, not many studies primarily examined the effects of sex. Akiyama et al. [1] found by multiple regression analysis that female sex was a predictor of internal hip rotation after THA, which was explained by soft tissue laxity in women. In the present study, internal rotation was not measured, but femoral torsion (defined by the angle between the femoral neck axis/femoral stem axis and the posterior condylar axis) was compared. During surgery, femoral torsion is set at 15° ± 10° according to Lewinnek [24], and it seemed that surgeons were successful with this, because a decrease in femoral torsion from 20° to 13° was observed in women.

Assessment of GO is an important part of THA planning. In this study, GO was defined as the sum of FO and AO. This combined measurement takes into account the changes caused by implant design, stem positioning as well as the changes in the acetabular center of rotation caused by cup implantation [26]. Mahmood et al. [28] found that after THA one-third of the patients had a reduced GO (< 5 mm), one-third was restored and one-third of the patients had an increased GO (> 5 mm). In our clinic, the goal of cup positioning is always to reach the original cup bottom during the milling process (lateral edge of the pelvic teardrop). Because of this medialization, GO must be compensated by increasing FO. It is believed that creating an ideal and usually longer offset improves the lever arm of the hip abductors and decreases the rate of polyethylene wear [30]. Specifically in women, implantation of a hip arthroplasty resulted in a significantly greater increase in FO. This may be attributed to the standardized prosthesis design, as no gender-specific prosthesis models are available. The AO was significantly more reduced in men than in women in our study, which may be due to the fact the more bone mass was removed in the acetabular base during the milling process.

The most notable change in leg alignment after THA is the decrease in HKA in all patients, but especially in women. The increase in FO in women seems to be directly related to the decrease in valgus inclination [41]. This must be taken into account when implanting the hip endoprosthesis, as an excessive increase in FO must be avoided to prevent pathologic varus malalignment of the leg axis. Several studies have shown that lower limb malalignment has an impact on knee OA by altering the distribution of loading stress and thus accelerating cartilage degeneration [9, 32]. Boissonneault et al. [4] investigated whether sex differences in hip and pelvic geometry parameters were associated with the presence of compartment-specific knee OA. They found that lateral knee OA was associated with reduced FO, greater hip center height, and greater NSA angle, whereas knees with medial OA were associated with smaller NSA. Particularly smaller women have a shorter femoral neck and thus a lower FO.

In addition, patients with primary hip OA are known to have a higher risk for developing knee OA in the contralateral leg [21, 22]. Contralateral knee OA likely originates from asymmetries in joint loading that occur early in the development of hip disease [20, 38].

## Limitations

This study has several limitations. First, this study was part of a large prospective study, so we could not control enrollment by sex and etiology of hip OA. This resulted in a small number of patients and an uneven distribution of patients among subgroups. In particular, for the comparisons between OA types, the post hoc calculated power was low (0.24). The power achieved for comparisons between the sexes was acceptable (0.76) when calculated as the mean across all calculated parameters (including femoral torsion). To improve the reproducibility and transferability and considering the subjectivity of implant placement, a multi-center study is desirable.

In addition, the calculation of clinical parameters in EOS is subject to a degree of uncertainty. Several studies have shown that EOS is a reliable and accurate system for assessing lengths and angles of the lower leg [13, 40]. Although rotational parameters such as femoral torsion show comparable results with standard CT measurements, it should be noted that inter-user reproducibility errors are quite large for both modalities [5, 29].

In this study, dysplasia patients were identified based on a center–edge angle < 35°. This is a rough classification, as others have expanded this classification into dysplasia < 25°, normal 25°–39°, and pincer impingement > 39° [17]. Our patient group was too small to divide into more than two groups. Future studies should include more patients to divide patients according to this more accurate classification.

## Conclusions

In the present study, THA was found to have a greater effect on leg axis in women than in men. Surgeons should consider the effects of THA on varus/valgus alignment of the leg in patients with hip OA. In future work, prospective studies with long follow-up (5–10 years) should investigate whether THA-related changes in leg alignment are also clinically relevant and lead to premature medial or lateral femoral knee OA on the ipsilateral side.

## Supplementary Information

Below is the link to the electronic supplementary material.

Supplementary file1 (DOCX 29 KB)

## Data Availability

De-identified subject data have been deposited in the Zenodo repository (https://doi.org/10.5281/zenodo.6802660).
